# The shift of expenditure of medical service for diagnosis and treatment of colorectal cancer from 2005 to 2014: a hospital-based retrospective survey in Guangxi

**DOI:** 10.3389/fpubh.2025.1668589

**Published:** 2025-11-05

**Authors:** Wenjie Liang, Haiyan Lu, Kaiyong Huang, Li Yang

**Affiliations:** ^1^Department of Social Medicine, School of Public Health, Guangxi Medical University, Nanning, Guangxi, China; ^2^Department of Occupational and Environmental Health, School of Public Health, Guangxi Medical University, Nanning, Guangxi, China

**Keywords:** colorectal neoplasms, medical cost, temporal trend, Guangxi, hospital-based study

## Abstract

**Purpose:**

Medical expenditures for colorectal cancer (CRC) in China have risen substantially, with significant geographic disparities. This study aimed to comprehensively assess the level, temporal trends, and influencing factors of medical expenditures per patient, per clinical visit, and per day among CRC patients in Guangxi from 2005 to 2014.

**Methods:**

A retrospective, hospital-based survey was conducted in 2015 at a Class A tertiary cancer hospital in Nanning, Guangxi. The study included patients newly diagnosed with primary CRC between January 1, 2005, and December 31, 2014. Data on demographics, clinical characteristics, and detailed medical costs were extracted from electronic medical records. All expenditure values were adjusted for inflation to 2011 Chinese Yuan (CNY) using China’s healthcare-specific Consumer Price Index. Statistical analyses included descriptive statistics, non-parametric tests, Spearman correlation, and binary logistic regression.

**Results:**

A total of 1,029 eligible CRC patients were analyzed. The median medical expenditures were CNY 50,540 per patient, CNY 24,618 per clinical visit/admission, and CNY 1,410 per day. From 2005 to 2014, these expenditures increased by factors of 1.63, 1.13, and 2.44, respectively, with annual growth rates of 5.59, 1.41, and 10.42%. Notably, the annual per-patient medical expense was 2.2 to 3.3 times the per-capita GDP of Guangxi during the same period. Drug fees constituted the largest proportion of total costs (61.22%), followed by laboratory test fees (12.27%) and inspection fees (7.58%). Subgroup and regression analyses revealed that treatment regimen, number of clinical visits/admissions, and length of hospitalization were significant factors associated with higher costs. A high proportion of patients (59.87%) were diagnosed at an advanced stage (III-IV).

**Conclusion:**

Medical expenditures for CRC diagnosis and treatment in Guangxi during the study period were substantial and increased rapidly, imposing a significant economic burden. The cost structure was heavily dominated by pharmaceutical expenses. The findings underscore the need for policies promoting early detection and efficient resource allocation. This study provides a critical baseline for evaluating the impact of subsequent healthcare reforms and informs future health economic research in Guangxi and similar regions.

## 1 Introduction

Colorectal cancer (CRC) remains a significant global public health challenge. While the global age-standardized mortality rate for CRC has declined, the age-standardized incidence rate has continued to rise in most countries (1). According to GLOBOCAN 2020 estimates, CRC accounted for over 1.9 million new cases and 935,000 deaths worldwide in 2020, ranking third in incidence and second in mortality among all cancers (2). Furthermore, the incidence of CRC among adolescents and young adults is increasing (3–5), and CRC was ranked fourth among the five cancers with the highest absolute disability-adjusted life-year burden in males aged 15–39 years (6). At the same time, survival trends for CRC have generally improved (7), a progress in which early detection through cancer screening has played an instrumental role (8, 9).

Additionally, three distinct patterns have been described regarding trends in CRC incidence and mortality across different countries: first, a concurrent increase in both incidence and mortality over the past decade; second, rising incidence accompanied by declining mortality; and third, a decrease in both measures (10, 11). China aligns with the first pattern. According to the Global Cancer Observatory, an estimated 550,628 new CRC cases and 283,751 deaths occurred in China in 2020, making CRC the second most common and fifth deadliest cancer nationwide (12). From 1990 to 2017, both CRC incidence and mortality exhibited consistent upward trends among both sexes (13). Furthermore, significant geographic disparities in cancer rates were observed across regions (13). Survival among CRC patients in China has gradually improved, with more substantial gains in rural compared to urban areas (7, 14). For example, in Guangxi Zhuang Autonomous Region in southwestern China, age-standardized incidence and mortality rates of CRC declined from 2013 to 2016 in both urban and rural populations, with a more pronounced decrease in rural regions (15–18).

Medical expenditures in China have risen substantially over the past three decades (19–22). Among all cancers, spending on CRC treatment in specialized cancer hospitals saw the highest increase between 2011 and 2015, rising by 117.0% (23). Of this total, medical costs accounted for 91.7% of the overall expenditure for CRC diagnosis and treatment (19). In addition, medical expenses for CRC exhibited significant geographic variation across different study sites, provinces, and regions (19, 20, 22).

A systematic review covering 1996–2015 (20) and two hospital-based multicenter surveys conducted between 2002 and 2014 (19, 22) on CRC medical expenses in China did not incorporate data from Guangxi. Although the most recent multicenter retrospective survey (24) included Guangxi, it only reported an overall average medical expenditure across all sites and did not conduct subgroup analyses by province. To date, no study has specifically evaluated medical expenses or long-term trends in CRC diagnosis and treatment within Guangxi. The present analysis aims to assess the level and trends in medical expenditure among CRC patients in Guangxi from 2005 to 2014, thereby establishing a valuable reference for understanding regional trends and supporting health-economic decisions.

## 2 Patients and methods

### 2.1 Study site and participants

This survey was conducted at a Class A tertiary specialized cancer hospital in Nanning, the capital city of Guangxi. The inclusion criteria were as follows: (1) patients whose first hospital visit for CRC diagnosis and/or treatment occurred between 1 January 2005 and 31 December 2014; (2) patients who received CRC therapy at the study hospital; and (3) patients whose complete clinical information could be captured, particularly details on cancer staging and pathological diagnosis. Patients who had only visited the hospital for diagnosis, post-treatment follow-up, or outpatient radiotherapy within the study period were excluded. Patients were not contacted directly, and information was obtained from their medical records in the study hospital (24, 25). The clinical stage of cancer was defined utilizing the TNM classification by the American Joint Committee on Cancer (AJCC) and the Union for International Cancer Control (UICC).

The survey was approved by the Institutional Review Board of the cancer hospital at the Chinese Academy of Medical Sciences (which waived the need to obtain informed consent from participants because of the retrospective and noninterventional design of this survey) as a component of a broader screening program (program 15–071/998).

**Figure 1:**
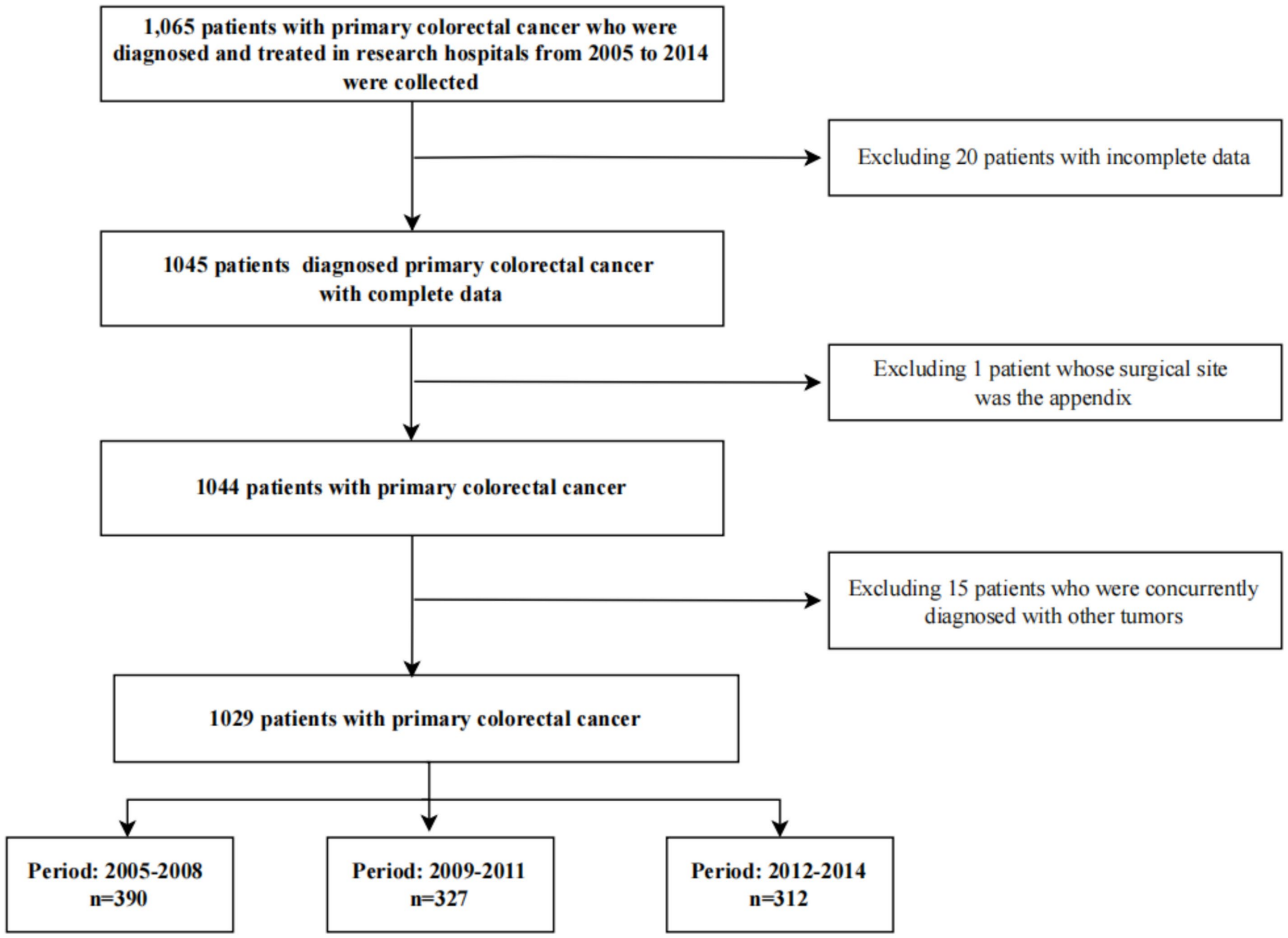
Patient selection flowchart.

The patient selection process is illustrated in Figure 1. Initially, 1,065 patients diagnosed with primary colorectal cancer between 2005 and 2014 were identified from the hospital’s electronic medical records. Subsequently, a multi-step exclusion criteria was applied to ensure data quality and homogeneity of the study cohort. First, 20 patients with incomplete key data (e.g., missing cost records or staging information) were excluded. Second, one patient whose surgical site was the appendix was removed to maintain consistency in tumor localization. Finally, 15 patients who were concurrently diagnosed with other primary malignancies were excluded to avoid potential confounding effects on medical expenditure analysis. After these exclusions, the final analytical cohort comprised 1,029 eligible patients with primary colorectal cancer, who were then stratified into three calendar periods for analysis: 2005–2008 (*n* = 390), 2009–2011 (*n* = 327), and 2012–2014 (*n* = 312).

### 2.2 Data acquisition and quality control

We included at least 100 patients each year from 2005 to 2014. We retrieved the date of the first clinical visit of each patient to the study hospital. We defined the first patients diagnosed in May 2005, June 2006, July 2007, August 2008, September 2009, October 2010, November 2011, December 2012, March 2013, and April 2014 as the first participants in their respective years (January and February were not considered since the fewest number of patients were hospitalized for diagnosis and treatment around the Spring Festival in China). Subsequently, we collected each eligible case whose date of clinical visit was after that of the first participant, until the figure of 100 or more patients was reached. A simplified form was used to classify patients with primary and nonprimary CRC. Patients with nonprimary CRC were excluded; those with primary CRC were administered a survey via a structured case report form (CRF), which was provided by the Chinese National Cancer Center (CNCC). The following data were collected: (1) basic demographic information, including hospital ID, name, gender, age, insurance status, occupation, etc.; (2) clinical information, such as clinical stage, pathological diagnosis, disease metastasis, main diagnosis, etc.; (3) details related to the expenditure, including the date of the first visit, hospitalization days, overall expenditure, component of expense, etc.

Our team was managed by an individual who had attended a training course organized by the CNCC in Beijing. Six postgraduate students from the school of public health in the Guangxi medical university were recruited as investigators and then trained and examined; only those who passed the examination became qualified investigators. There were specific staff who worked in the study hospital to assist with data collection. The investigators worked in pairs; one investigator retrieved data from the electronic information system of the hospital and recorded this information on the paper-based CRF, while the other investigator rechecked the data. Data of the CRFs were entered onto the CNCC’s purpose-built database (EpiData 3.1, Odense, Denmark); an investigator double-entered the CRF data, then the data were subsequently submitted to the CNCC system. The CNCC checked the data which we submitted and raised suggestions about the unqualified data; we supplemented or modified the information based on these.

### 2.3 Data analysis

All statistical analyses were performed using SPSS version 20 (IBM, D0EJNLL, New York, United States). The records of 36 patients were excluded from analysis due to missing data. Based on the timing of the announcement of the official government reform plan, health reform in China can be broadly classified into two phases: Phase One (2009 to 2011) and Phase Two (2012 onward) (26). Accordingly, we stratified the study period as follows: 2005–2008; 2009–2011; and 2012–2014.

The expenditure data from this study and other cited studies were converted into the 2011 Chinese yuan (CNY; 1 CNY = 0.155 US dollar), which was inflated using the year-specific healthcare consumer price index of China obtained from the official website of the National Bureau of Statistics of China (27). Specially, expenditure data converted to 2011 values were consistent with a previous national study (24), Shi Jufang aimed to conduct direct comparisons with the national average, the average levels of different regions, the average levels of different hospital types, the average levels of different hospital, and so on.

In addition, the gross domestic product per capita was converted into the 2011 Chinese yuan by the year-specific gross domestic product per capita index. Demographic and clinical characteristics were described using proportions; age at diagnosis was described using the mean value; the number of clinical visits/admissions, the hospitalization days, and all expenses were described using the median. The expenditure data were compared within subgroups of characteristics using the nonparametric test; the Chi-square test or Fisher exact test were performed to detect any differences in clinical and demographic data among three calendar periods, as appropriate. Temporal trends of all medical expenses, the number of clinical visits/admissions, and hospitalization days from 2005 to 2014 were investigated using Spearman correlation analysis. The correlation analysis between medical expenses and the gross domestic product per capita was also investigated using Spearman correlation analysis. We transformed the medical costs into binary categorical variables based on the median. The medical cost that was lower than the median was classified as the ‘low-cost group,’ and that which was equal to or higher than the median was classified as the ‘high-cost group.’ The influencing factors of medical expenditure were identified utilizing the binary logistic regression analysis; to achieve an optimal goodness-of-fit test of the binary logistic regression model, the number of clinical visits/admissions and inpatient days were excluded from the analysis of the medical expense per clinical visit/admission. The AAPC of medical expenditure per patient, per clinical visit/admission, and per day was calculated; the AAPC of hospitalization days was also computed. The medical expenditure was evaluated in a whole period (2005–2014) and three calendar periods: 2005–2008, the period before the healthcare reform; 2009–2011, the first phase of the healthcare reform; and 2012–2014, the second phase of the healthcare reform. All statistical tests were two-tailed, and *p*-values less than 0.05 were considered statistically significant.

### 2.4 Sensitivity analysis for sample representativeness

To assess the potential for selection bias and evaluate the robustness of our primary findings regarding sample representativeness, we conducted sensitivity analyses using subgroup consistency tests. This approach tests whether the key temporal trends in medical expenditures were consistent across diverse patient strata, which would indicate that the overall findings were not overly sensitive to the specific composition of our single-center sample. We stratified the study cohort by key demographic and clinical characteristics, including gender (male, female), age group at diagnosis (<45, 45–54, 55–64, ≥65 years), health insurance status (insured, uninsured), cancer stage at diagnosis (I-II, III-IV), and primary treatment regimen (chemotherapy alone, surgery alone, surgery combined with chemotherapy). For each subgroup, we calculated the average AAPC from 2005 to 2014 for the three primary expenditure outcomes: medical expenditure per patient, per clinical visit/admission, and per day. The consistency of the AAPC estimates and their directions (increasing or decreasing) across the various subgroups was then qualitatively evaluated. Robust and generalizable trends would be evidenced by similar AAPC values and uniform directional changes in expenditure outcomes across the majority of patient strata.

## 3 Results

### 3.1 Demographic and clinical characteristics of patients

In total, records of 1,065 patients diagnosed with primary CRC from 2005 to 2014 in our study hospital were collected, of which 1,029 were available. Male patients constituted 60.64% of the total (male-to-female ratio: 1.54). The mean age at diagnosis was 57.25 years, with 34.31% of them being 65 years or more. In all, 28.18% of patients had received treatment at other hospitals. The most frequently observed stage at diagnosis was the advanced stage (59.87% of stage III–IV), and the least was stage I (3.89%). The proportions of rectal and colon cancer were similar. Adenocarcinoma was the most common histological type of cancer (92.71%). The majority of patients (72.30%) were diagnosed without metastatic disease. Most patients received surgery combined with chemotherapy. Approximately half of the cases had only one clinical visit/hospitalization. The median of inpatient days was 35 days. Among the three calendar periods, there were differences in distributions of health insurance status, number of clinical visits/admissions, or therapeutic patterns. The details are displayed in Table 1.

**Table 1:** Characteristics of colorectal cancer patients in 2005–2014, Guangxi.

Characteristics	2005–2014	Calendar periods			
		2005–2008	2009–2011	2012–2014	*p*-value^▲^
Overall, n (%)	1,029 (100.00)	390 (37.90)	327 (31.78)	312 (30.32)	
Gender, n (%)					0.315
Male	624 (60.64)	247 (63.33)	189 (57.80)	188 (60.26)	
Female	405 (39.36)	143 (36.67)	138 (42.20)	124 (39.74)	
Age at diagnosis (year), Mean ± SD	57.25 ± 13.44	56.24 ± 13.75	58.69 ± 13.62	57.00 ± 12.76	**0.048**
Age at diagnosis (year)					0.046
﹤45	197 (19.14)	83 (21.28)	59 (18.04)	55 (17.63)	
45–54	210 (20.41)	80 (20.51)	54 (16.51)	76 (24.36)	
55–64	269 (26.41)	101 (25.90)	81 (24.77)	87 (27.88)	
≥65	353 (34.31)	126 (32.31)	133 (40.67)	94 (30.13)	
Health insurance status, n (%)					**<0.001**
Urban employee basic medical insurance	232 (22.55)	60 (15.38)	80 (24.46)	92 (29.49)	
New rural cooperative medical scheme	250 (24.30)	6 (1.54)	113 (34.56)	131 (41.99)	
No insurance	449 (43.63)	298 (76.41)	89 (27.22)	62 (19.87)	
Others	98 (9.52)	26 (6.67)	45 (13.76)	27 (8.65)	
Received treatment at other hospital, n (%)					0.109
No	673 (65.40)	230 (58.97)	217 (66.36)	226 (72.44)	
Yes	290 (28.18)	117 (30.00)	93 (28.44)	80 (25.64)	
Not reported*	66 (6.42)	43 (11.03)	17 (5.20)	6 (1.92)	
Stage at diagnosis, n (%)					0.123
I	40 (3.89)	7 (1.79)	16 (4.89)	17 (5.45)	
II	312 (30.32)	117 (30.00)	101 (30.89)	94 (30.13)	
III	331 (32.17)	139 (35.64)	102 (31.19)	90 (28.85)	
IV	285 (27.70)	116 (29.74)	90 (27.52)	79 (25.32)	
Not reported*	61 (5.92)	11 (2.82)	18 (5.5)	32 (10.26)	
Tumor localization, n (%)					0.618
Rectum	530 (51.51)	200 (51.28)	165 (50.46)	165 (52.88)	
Colon	469 (45.58)	176 (45.13)	158 (48.32)	135 (43.27)	
Others	30 (2.92)	14 (3.59)	4 (1.22)	12 (3.85)	
Histologic type, n (%)					0.734
Adenocarcinoma	954 (92.71)	359 (92.05)	306 (93.58)	289 (92.63)	
Others	75 (7.29)	31 (7.95)	21 (6.42)	23 (7.37)	
Metastasis at diagnosis, n (%)					0.913
Yes	217 (21.09)	80 (20.51)	71 (21.71)	66 (21.15)	
No	744 (72.30)	264 (67.69)	244 (74.62)	236 (75.64)	
Not reported*	68 (6.61)	46 (11.79)	12 (3.67)	10 (3.21)	
Number of clinical visits per patient, Median (*P*_25,_ *P*_75_)	2 (1, 3)	1 (1, 3)	1 (1, 3)	1 (1, 4)	**<0.001**
Number of clinical visits per patient, n (%)					**<0.001**
1	463 (45.00)	202 (51.79)	167 (51.07)	94 (30.13)	
2	160 (15.55)	59 (15.13)	51 (15.60)	50 (16.03)	
3	166 (16.13)	66 (16.92)	40 (12.23)	60 (19.23)	
4+	240 (23.32)	63 (16.15)	69 (21.10)	108 (34.62)	
Treatment regimens, n (%)					**<0.001**
Chemotherapy alone	251 (24.39)	90 (23.08)	85 (25.99)	76 (24.36)	
Surgery alone	341 (33.14)	108 (27.69)	136 (41.59)	97 (31.09)	
Surgery combined with chemotherapy	390 (37.90)	179 (45.90)	91 (27.83)	120 (38.46)	
Others	47 (4.57)	13 (3.33)	15 (4.59)	19 (6.09)	
Number of inpatient days per patient, Median (*P*_25,_ *P*_75_)	35 (23, 59)	43 (25, 80)	28 (21, 46)	34 (24, 50)	**<0.001**

^▲^Multiple comparisons were performed among 2005–2008, 2009–2011, 2012–2014; *Data were omitted from statistical analysis. SD, standard deviation; P_25_, lower quartile; P_75_, upper quartile; Guangxi, Guangxi Zhuang Autonomous Region. Bold values indicate statistical significance (*p* < 0.05).

### 3.2 Medical expenditure per patient

#### 3.2.1 Medical expenditure per patient in each subgroup

The overall median medical expenditure per patient was 50,540 CNY. Subgroup analysis revealed that costs differed significantly based on the number of clinical visits and treatment regimens (Table 2). Moreover, Table 2 also showed that the subgroups with discrepancies in the medical expense per patient were not completely consistent in the three calendar periods. For instance, the medical spending per patient differed by stage at diagnosis, metastasis at diagnosis, number of clinical visits/admissions, and treatment regimens in 2012–2014, but it varied by health insurance status, number of clinical visits, and treatment regimens in 2005–2008. The binary logistic model showed that the medical cost per patient was positively associated with health insurance status, metastasis at diagnosis, number of clinical visits, inpatient days, and treatment regimens (Table 3).

**Table 2:** The medical expense per patient for colorectal cancer cases in 2005–2014, Guangxi.

Characteristics	Medical expenditure per patient (CNY), Median (IQR)							
	2005–2014	*p*-value	2005–2008	*p*-value	2009–2011	*p*-value	2012–2014	*p*-value
Overall	50,540 (38643)	NA	39,149 (33497)	NA	47,842 (30192)	NA	66,688 (36159)	NA
Gender		0.711		0.720		0.519		0.623
Male	39,149 (33497)		38,638 (34223)		49,777 (36408)		66,654 (34739)	
Female	56,543 (36375)		39,954 (33519)		47,514 (27019)		66,919 (37613)	
Age at diagnosis (year)		0.268		0.612		0.624		0.106
<45	48,751 (37143)		42,176 (38500)		47,452 (32719)		63,136 (41079)	
45–54	54,758 (43901)		43,503 (32744)		46,385 (44445)		73,560 (41767)	
55–64	50,519 (37145)		40,451 (35277)		45,428 (23990)		66,768 (30940)	
≥65	48,623 (39138)		34,555 (26630)		51,539 (28706)		64,648 (38620)	
Health insurance status		**<0.001**		**0.003**		**0.001**		0.279
Had insurance	57,210 (34565)		48,090 (37899)		51,854 (31484)		64,915 (34409)	
No insurance	42,494 (34167)		36,349 (32959)		44,343 (28335)		71,192 (41550)	
Stage at diagnosis		0.052		0.197		**0.008**		**0.001**
I and II	49,744 (31876)		34,361 (26911)		47,170 (21871)		69,763 (34391)	
III	52,892 (38105)		39,159 (32170)		52,890 (32727)		72,498 (39624)	
IV	47,190 (47268)		43,692 (43932)		43,818 (52623)		56,229 (41705)	
Tumor localization		0.318		0.842		0.328		0.856
Rectum	52,792 (36826)		38,595 (36013)		51,539 (28036)		66,567 (35492)	
Colon	48,501 (40251)		39,149 (30410)		46,847 (31418)		63,706 (36757)	
Histologic type		0.722		0.707		0.756		0.617
Adenocarcinoma	50,529 (38711)		39,219 (33346)		47,924 (30716)		66,748 (36841)	
Others	51,655 (42510)		37,230 (46200)		47,452 (33162)		66,567 (26981)	
Metastasis at diagnosis		0.440		0.201		0.055		**0.018**
Yes	48,132 (51089)		42,726 (53082)		46,207 (55175)		60,261 (46577)	
No	51,899 (36148)		37,139 (31601)		48,813 (26542)		68,645 (33442)	
Number of clinical visits per patient		**<0.001**		**<0.001**		**<0.001**		**<0.001**
1	39,625 (24675)		30,178 (18308)		43,453 (15447)		47,704 (22887)	
2	51,062 (34161)		45,064 (29475)		48,623 (29932)		62,198 (34317)	
3	63,716 (35410)		54,415 (34572)		66,113 (47301)		70,157 (26059)	
4+	77,359 (39204)		63,695 (34139)		73,880 (45859)		84,784 (31412)	
Treatment regimens		**<0.001**		**<0.001**		**<0.001**		**<0.001**
Chemotherapy alone	33,812 (42045)		29,717 (37763)		25,941 (31727)		50,031 (47236)	
Surgery alone	44,830 (25856)		29,440 (16311)		46,475 (16019)		56,286 (29491)	
Surgery combined with chemotherapy	64,072 (39212)		48,287 (28450)		72,259 (44085)		76,613 (29620)	

CNY, China Yuan; IQR, interquartile range; Guangxi, Guangxi Zhuang Autonomous Region. Bold values indicate statistical significance (*p* < 0.05).

**Table 3:** Binary logistic regression between the medical expense and variables.

Variables	B	*p*-value	OR (95% CI)
The medical expense per patient			
Intercept	−4.70	**<0.001**	0.009
Health insurance status (with VS without)	−1.40	**<0.001**	0.25 (0.16–0.39)
Metastasis at diagnosis (with VS without)	0.69	**0.013**	2.00 (1.16–3.43)
Number of clinical visits per patient (2 times VS 1 time)	1.22	**<0.001**	3.38 (1.83–6.26)
Number of clinical visits per patient (3 times VS 1 time)	2.33	**<0.001**	10.28 (5.07–20.84)
Number of clinical visits per patient (4 + times VS 1 time)	3.47	**<0.001**	32.13 (14.03–73.57)
Treatment regimen (Surgery alone VS Chemotherapy alone)	3.16	**<0.001**	23.64 (10.25–54.51)
Treatment regimen (Surgery combined with chemotherapy VS Chemotherapy alone)	2.48	**<0.001**	11.99 (6.05–23.73)
Inpatient days (25–34 days VS < 25 days)	0.63	**0.045**	1.88 (1.01–3.49)
Inpatient days (35–58 days VS < 25 days)	2.17	**<0.001**	8.75 (4.60–16.64)
Inpatient days (≥59 days VS < 25 days)	4.55	**<0.001**	94.29 (38.41–231.49)
The medical expense per clinical/admission			
Intercept	−1.8	**<0.001**	0.165
Sex (Male VS Female)	−0.42	**0.017**	0.66 (0.46–0.93)
Age at diagnosis (45–54 years VS < 45 years)	0.40	0.147	1.49 (0.86–2.55)
Age at diagnosis (55–64 years VS < 45 years)	0.33	0.191	1.40 (0.84–2.30)
Age at diagnosis (≥65 years VS < 45 years)	0.79	**0.001**	2.21 (1.35–3.60)
Treatment regimen (Surgery alone VS Chemotherapy alone)	3.04	**<0.001**	20.89 (12.45–35.06)
Treatment regimen (Surgery combined with chemotherapy VS Chemotherapy alone)	1.09	**<0.001**	2.97 (1.87–4.70)
The medical expense per day			
Intercept	0.06	0.858	1.06
Age at diagnosis (45–54 years VS < 45 years)	0.12	0.666	1.13 (0.64–1.97)
Age at diagnosis (55–64 years VS < 45 years)	0.22	0.402	1.24 (0.74–2.07)
Age at diagnosis (≥65 years VS < 45 years)	0.63	**0.013**	1.88 (1.14–3.07)
Health insurance status (with VS without)	−1.99	**<0.001**	0.14 (0.10–0.20)
Number of clinical visits per patient (2 times VS 1 time)	0.68	**0.015**	1.98 (1.14–3.44)
Number of clinical visits per patient (3 times VS 1 time)	0.81	**0.007**	2.25 (1.25–4.05)
Number of clinical visits per patient (4 + times VS 1 time)	1.32	**<0.001**	3.76 (2.09–6.74)
Treatment regimen (Surgery alone VS chemotherapy alone)	1.84	**<0.001**	6.32 (3.77–10.57)
Treatment regimen (Surgery combined with chemotherapy VS chemotherapy alone)	0.61	**0.011**	1.84 (1.15–2.94)
Inpatient days (25–34 days VS < 25 days)	−0.39	0.132	0.68 (0.40–1.13)
Inpatient days (35–58 days VS < 25 days)	−1.18	**<0.001**	0.31 (0.18–0.53)
Inpatient days (≥59 days VS < 25 days)	−1.87	**<0.001**	0.16 (0.08–0.29)

B, regression coefficient; OR, Odds Ratio; 95%CI, 95% confidence interval; Guangxi, Guangxi Zhuang Autonomous Region. Only statistically significant parameter (*p* < 0.05) in the final binary logistic regression model are show. Bold values indicate statistical significance (*p* < 0.05).

**Figure 2:**
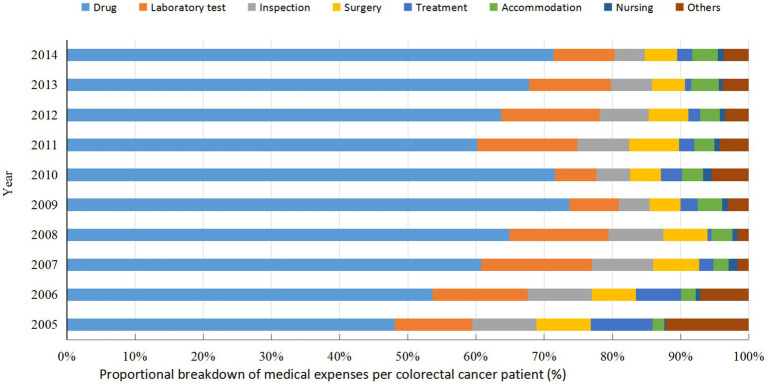
Proportional distribution of medical expenses per colorectal cancer patient in 2005–2014, Guangxi.

Further analysis of the medical costs for colorectal cancer cases in Guangxi from 2005 to 2014 indicated that drug fees constituted the largest proportion of total expenses. As summarized in Table 4, drugs accounted for 61.22% of the overall costs during this period, followed by laboratory tests (12.27%) and inspection fees (7.38%). The remaining components—surgery, treatment, accommodation, nursing, and other fees—comprised 6.21, 3.88, 2.73, 0.75, and 5.35% of the total, respectively. The proportional structure of expenses is further illustrated in Figure 2, which displays the annual breakdown of medical expenditures per patient in stacked bar charts. The data visualization confirms that drug fees consistently represented the primary cost component across all years. A notable increase in the proportion of drug costs was observed from 2005 to 2009, while other categories, including laboratory tests, inspections, and surgery, maintained comparatively stable and lesser shares throughout the decade.

**Table 4:** Detailed breakdown of medical expenses per patient for colorectal cancer cases in 2005–2014, Guangxi.

Year	Total expenses (CNY, %)	Proportional breakdown of medical expenses per colorectal cancer patient (CNY, %)							
		Drug	Laboratory test	Inspection	Surgery	Treatment	Accommodation	Nursing	Others
2005	7,835,254 (100.00)	3,769,937 (48.12)	887,606 (11.33)	737,609 (9.41)	626,057 (7.99)	709,432 (9.05)	134,803 (1.72)	14,961 (0.19)	954,848 (12.19)
2006	8,265,712 (100.00)	4,434,033 (53.64)	1,148,932 (13.90)	780,102 (9.44)	536,464 (6.49)	544,305 (6.59)	177,802 (2.15)	54,039 (0.65)	590,036 (7.14)
2007	5,443,368 (100.00)	3,305,575 (60.73)	883,363 (16.23)	488,331 (8.97)	368,137 (6.76)	114,616 (2.11)	121,878 (2.24)	71,170 (1.31)	90,297 (1.66)
2008	4,311,653 (100.00)	2,797,187 (64.88)	628,499 (14.58)	346,479 (8.04)	277,679 (6.44)	25,494 (0.59)	131,324 (3.05)	31,711 (0.74)	73,280 (1.70)
2009	4,294,125 (100.00)	3,162,204 (73.64)	311,570 (7.26)	197,023 (4.59)	194,245 (4.52)	107,574 (2.51)	153,240 (3.57)	37,534 (0.87)	130,735 (3.04)
2010	3,252,857 (100.00)	2,329,497 (71.61)	196,153 (6.03)	159,437 (4.90)	149,316 (4.59)	100,047 (3.08)	100,603 (3.09)	39,986 (1.23)	177,817 (5.47)
2011	3,759,332 (100.00)	2,261,743 (60.16)	549,683 (14.62)	288,221 (7.67)	274,216 (7.29)	84,615 (2.25)	110,764 (2.95)	30,596 (0.81)	159,494 (4.24)
2012	4,024,452 (100.00)	2,563,154 (63.69)	582,239 (14.47)	289,009 (7.18)	232,662 (5.78)	70,232 (1.75)	115,916 (2.88)	30,810 (0.77)	140,430 (3.49)
2013	3,376,289 (100.00)	2,288,344 (67.78)	405,024 (12.00)	204,124 (6.05)	162,261 (4.81)	135,055 (0.97)	135,055 (4.00)	19,801 (0.59)	128,926 (3.82)
2014	3,660,224 (100.00)	2,612,138 (71.37)	326,217 (8.91)	162,956 (4.45)	172,534 (4.71)	135,644 (2.28)	135,644 (3.71)	31,280 (0.85)	135,989 (3.72)
Overall	48,223,266 (100.00)	29,523,811 (61.22)	5,919,287 (12.27)	3,653,290 (7.58)	2,993,571 (6.21)	1,872,537 (3.88)	1,317,030 (2.73)	361,888 (0.75)	2,581,852 (5.35)

#### 3.2.2 Temporal trend of the medical expenditure per patient

**Figure 3:**
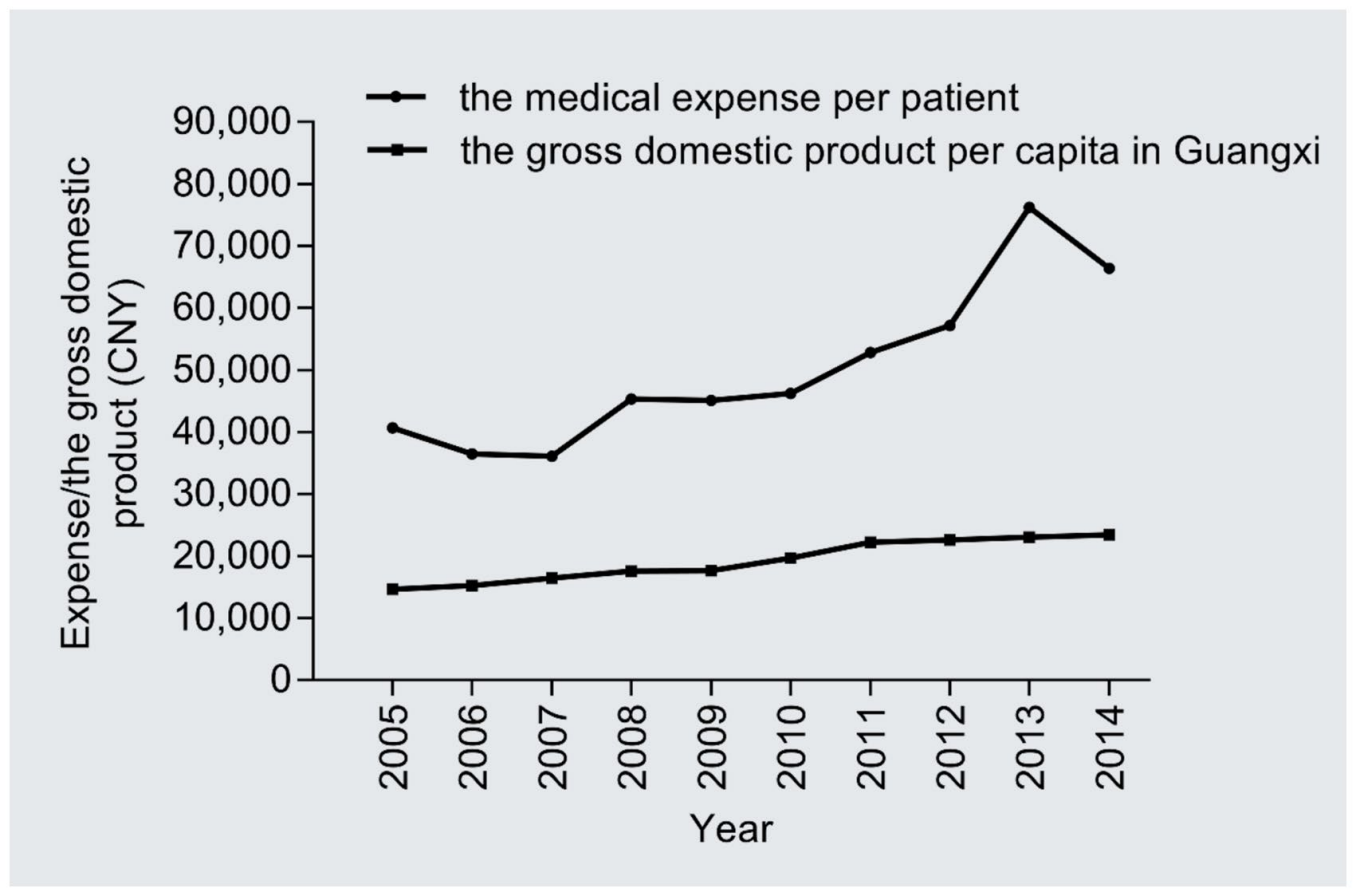
Temporal trend of the medical expenditure per patient.

The medical cost per patient increased notably from 40,706 CNY in 2005 to 66,413 CNY in 2014 (1.63 times; *r* = 0.35, 95%CI: 0.30–0.41, P trend<0.001), with an AAPC of 5.59% (Figure 3); the AAPC values were 3.66% in 2005–2008, 8.24% in 2009–2011, and 7.74% in 2012–2014. The gross domestic product per capita in Guangxi rose from 14,651 CNY in 2005 to 23,417 CNY in 2014 (1.59 times; *r* = 1.0, 95%CI:1.0–1.0, P trend<0.001), with an AAPC of 5.35% (Figure 3); the AAPC values were 6.31% in 2005–2008, 12.29% in 2009–2011, and 1.72% in 2012–2014. The medical expenses per patient were significantly positively associated with the gross domestic product of Guangxi (*r* = 0.93, 95%CI: 0.62–1.00, *p* < 0.001); more importantly, the annual medical expenses per patient were 2.20–3.30 times the annual gross domestic product per capita in Guangxi from 2005 to 2014.

### 3.3 Medical cost per clinical visit/admission

#### 3.3.1 Medical cost per clinical visit/admission in subgroups

As Table 5 shown, the average medical expense per clinical visit/admission was 24,618 CNY in 2005–2014, with a median clinical visit/admission of 2 times. The medical expenses per clinical visit/admission varied by age at diagnosis, number of clinical visits/admissions, and therapeutic regimens. In addition, subgroups with differences in medical expenses per clinical visit/admission were not fully coincident in the three calendar periods. For example, the medical spending per clinical visits/admissions varied by age at diagnosis, health insurance status, stage at diagnosis, metastasis at diagnosis, number of clinical visits, and treatment regimens in 2005–2008; whereas it differed by age at diagnosis, tumor localization, number of clinical visits, and treatment regimens in 2012–2014. The binary logistic model demonstrated sex, age at diagnosis, and therapeutic patterns were correlative with the medical cost per clinical visit/admission (Table 3).

**Table 5:** The medical expense per clinical visit/admission for colorectal cancer patients in 2005–2014, Guangxi.

Characteristics	Medical expense per clinical visit/admission (CNY), Median (IQR)							
	2005–2014	*p*-value	2005–2008	*p*-value	2009–2011	*p*-value	2012–2014	*p*-value
Overall	24,618 (25145)	NA	23,564 (18456)	NA	30,080 (29536)	NA	23,781 (25630)	NA
Gender		0.151		0.834		0.345		0.458
Male	23,785 (25380)		22,737 (18365)		27,906 (30849)		23,130 (24912)	
Female	27,514 (25637)		23,829 (18657)		33,855 (26933)		25,243 (28945)	
Age at diagnosis (year)		**<0.001**		**0.008**		**<0.001**		**0.001**
<45	19,945 (21258)		18,523 (19856)		19,889 (28180)		20,736 (24243)	
45–54	22,154 (22606)		22,331 (18552)		22,457 (24684)		20,758 (23439)	
55–64	23,114 (21033)		23,595 (17463)		23,611 (27088)		22,336 (15663)	
≥65	31,984 (27945)		27,650 (18314)		39,814 (28797)		33,129 (34225)	
Health insurance status		**<0.001**		**0.007**		**0.011**		0.860
Had insurance	27,236 (28220)		27,108 (26650)		33,481 (28674)		23,917 (26304)	
No insurance	22,628 (21487)		22,532 (17936)		25,196 (31724)		21,491 (24634)	
Stage at diagnosis		0.156		**0.020**		**0.001**		0.395
I and II	27,617 (25539)		22,379 (16578)		34,234 (25529)		23,819 (30304)	
III	24,095 (23575)		21,604 (17669)		36,382 (29803)		21,730 (17855)	
IV	22,839 (27276)		26,142 (26280)		18,949 (26701)		21,129 (22011)	
Tumor localization		0.787		0.257		0.954		**0.032**
Rectum	24,610 (24035)		22,295 (16648)		29,135 (28067)		25,463 (23351)	
Colon	25,037 (27242)		24,713 (20099)		31,652 (30582)		20,736 (30257)	
Histologic type		**0.040**		0.625		0.229		0.059
Adenocarcinoma	24,900 (25367)		23,595 (18690)		30,732 (28902)		24,114 (25628)	
Others	21,217 (20995)		22,136 (14528)		22,754 (30594)		18,493 (14949)	
Metastasis at diagnosis		0.796		**0.002**		**0.051**		0.641
Yes	24,114 (27141)		30,335 (29220)		22,182 (30281)		22,851 (21296)	
No	25,132 (24727)		22,246 (16029)		33,569 (28886)		24,089 (26575)	
No. of clinical visits per patient		**<0.001**		**<0.001**		**<0.001**		**<0.001**
1	39,625 (24674)		30,177 (18308)		43,453 (15447)		47,704 (22887)	
2	25,530 (17081)		22,532 (14737)		24,312 (14966)		31,099 (17158)	
3	21,238 (11803)		18,138 (11524)		22,038 (15767)		23,386 (8686)	
4+	15,070 (7248)		12,948 (6430)		13,785 (8066)		16,072 (6619)	
Treatment regimens		**<0.001**		**<0.001**		**<0.001**		**<0.001**
Chemotherapy alone	13,158 (13793)		14,153 (32096)		12,184 (10823)		13,941 (8784)	
Surgery alone	39,205 (20279)		27,554 (10669)		43,567 (15317)		45,382 (17341)	
Surgery combined with chemotherapy	21,599 (16693)		22,136 (18087)		22,328 (25288)		20,500 (9754)	

CNY, China Yuan; IQR, interquartile range; Guangxi, Guangxi Zhuang Autonomous Region. Bold values indicate statistical significance (*p* < 0.05).

#### 3.3.2 Temporal trend of the medical cost per clinical visit/admission

**Figure 4:**
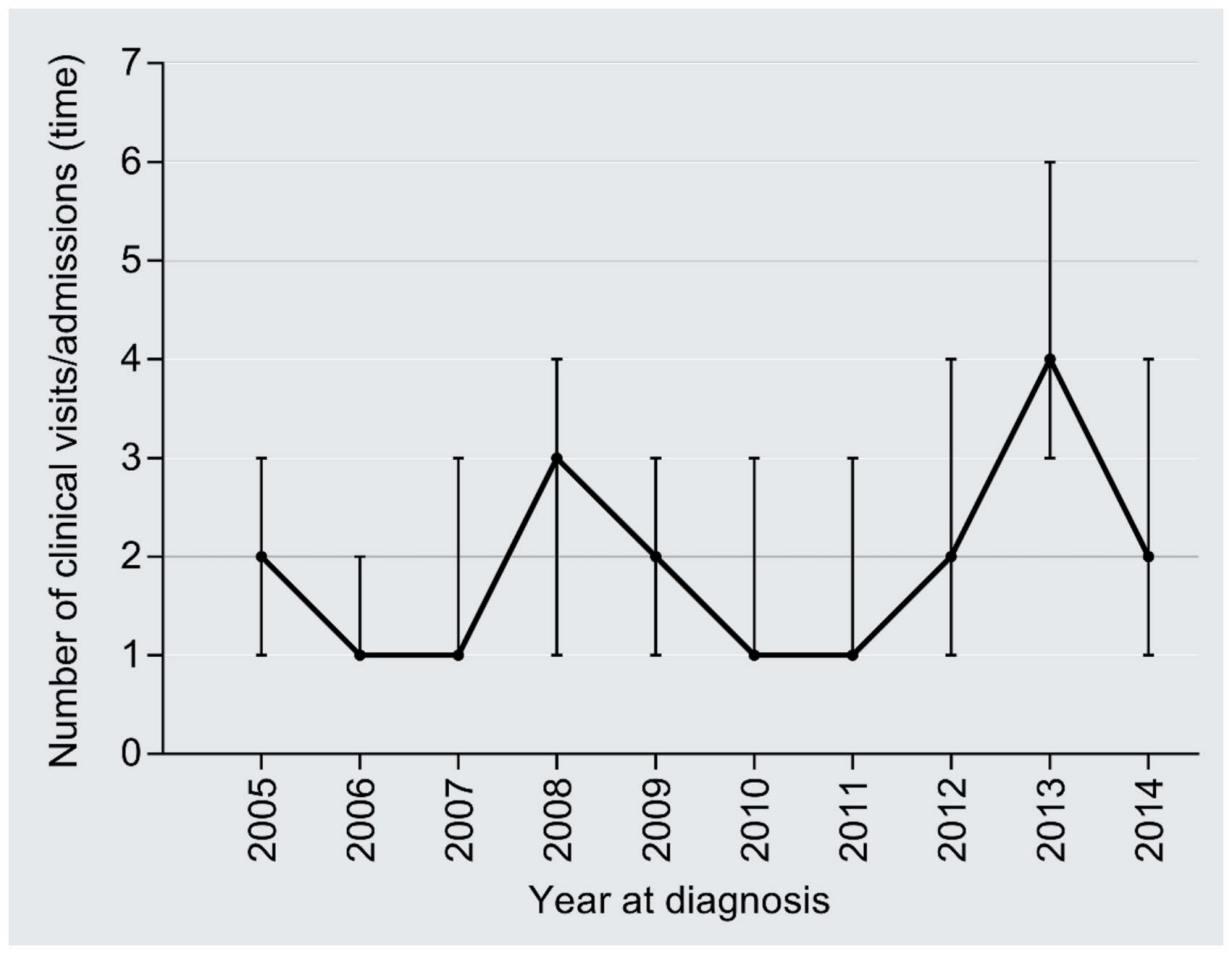
Temporal trend of median number of visits/hospitalizations per capita.

**Figure 5:**
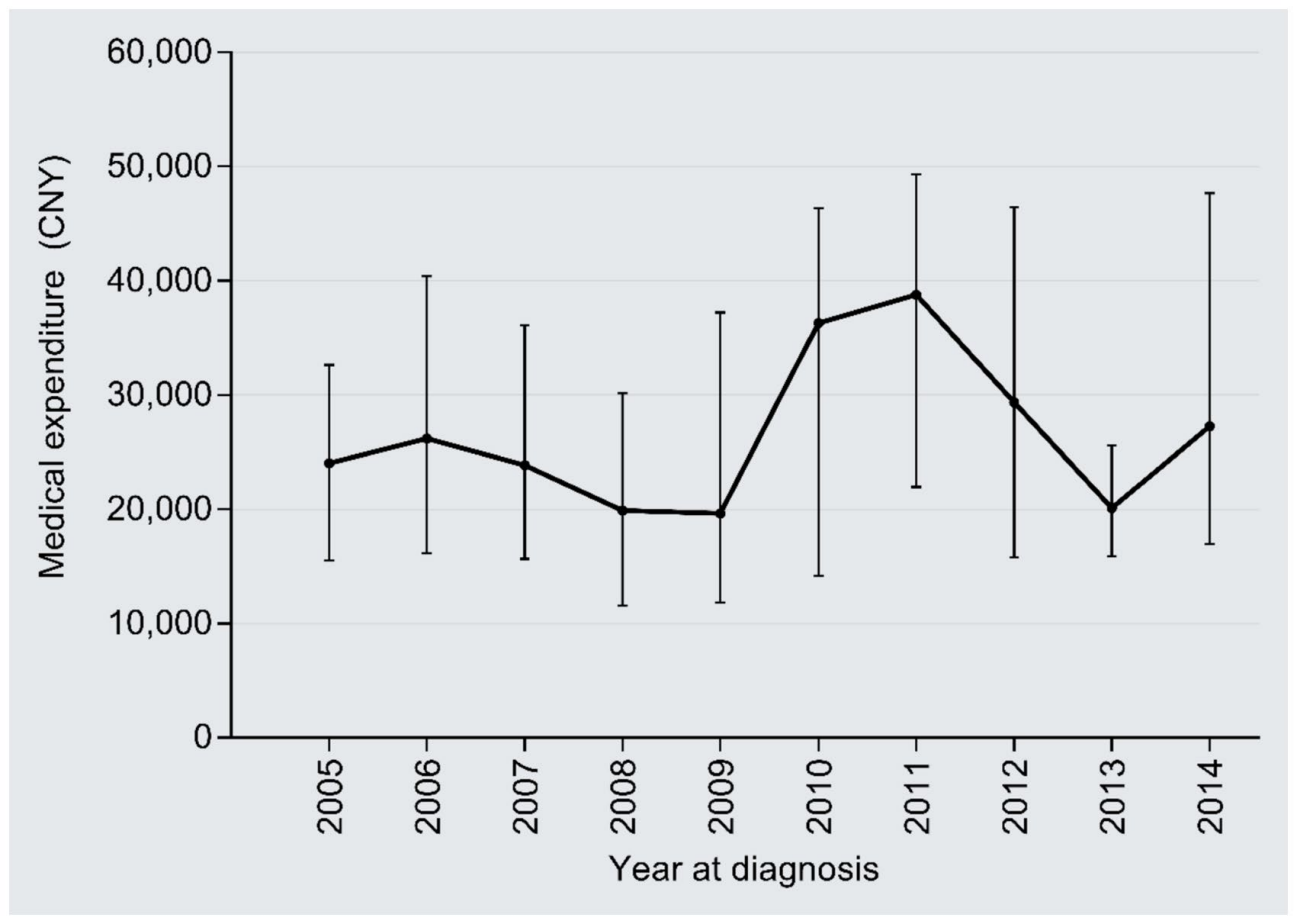
Temporal trend of the medical cost per clinical visit/admission.

The median number of clinical visits/admissions per patient showed a wavy increased trend over time in the study period (*r* = 0.09, 95%CI: 0.03–0.14, *P* trend = 0.006) (Figure 4). The medical expenditure per clinical visit/admission escalated from 24,026 CNY in 2005 to 27,255 CNY in 2014 (1.13 times; *r* = 0.09, 95%CI:0.03–0.14, *P* trend = 0.006), with an AAPC of 1.41% (Figure 5); the AAPC values were −6.08% in 2005–2008, 40.50% in 2009–2011, and −3.66% in 2012–2014.

### 3.4 Medical expense per day

#### 3.4.1 Medical expense per day in subgroups

The medical expenditure per day was 1,410 CNY in 2005–2014, with the median number of inpatient days being 32 days. The subgroup analysis found that the medical expenses per day differed by number of clinical visits/admission and treatment modalities (Table 6). Subgroups with disparities in medical expenses per day were not utterly accordant in each calendar period. As an example, the medical cost per day varied by gender, number of clinical visits, and treatment regimens in 2005–2008; however, this differed by age at diagnosis, stage at diagnosis, metastasis at diagnosis, number of clinical visits, and treatment patterns in 2012–2014. The binary logistic analysis indicated that age at diagnosis, health insurance status, stage at diagnosis, inpatient days, number of clinical visits/admissions, and treatment regimens were associated with the medical expenditures per day (Table 3).

**Table 6:** The medical expense per day for colorectal cancer patients in 2005–2014, Guangxi.

Characteristics	Medical expense per day (CNY), Median (IQR)							
	2005–2014	*p*-value	2005–2008	*p*-value	2009–2011	*p*-value	2012–2014	*p*-value
Overall	1,410 (1020)		868 (525)	NA	1,572 (803)	NA	1900 (815)	NA
Gender		0.998		**0.006**		0.357		0.608
Male	1,391 (1021)		902 (531)		1,479 (909)		1897 (811)	
Female	1,432 (1037)		809 (532)		1,644 (679)		1905 (817)	
Age at diagnosis (year)		**<0.001**		0.154		**<0.001**		**0.001**
<45	1,246 (904)		809 (439)		1,437 (700)		1787 (797)	
45–54	1,347 (935)		821 (538)		1,327 (603)		1827 (684)	
55–64	1,402 (1013)		912 (568)		1,553 (1098)		1871 (803)	
≥65	1,563 (1081)		953 (552)		1723 (805)		2,132 (809)	
Health insurance status		**<0.001**		0.713		**<0.001**		0.791
Had insurance	1,690 (858)		841 (568)		1,639 (726)		1910 (816)	
No insurance	1,044 (738)		868 (523)		1,360 (893)		1843 (862)	
Stage at diagnosis		**<0.001**		0.195		**0.002**		**0.002**
I and II	1,483 (1056)		883 (533)		1,645 (671)		2047 (700)	
III	1,386 (940)		902 (477)		1,587 (724)		1871 (866)	
IV	1,248 (882)		806 (599)		1,338 (820)		1,672 (608)	
Tumor localization		0.364		0.848		0.123		0.869
Rectum	1,392 (987)		862 (471)		1,505 (740)		1871 (902)	
Colon	1,430 (1011)		882 (575)		1,652 (977)		1900 (763)	
Histologic type		**0.027**		0.675		**0.007**		0.634
Adenocarcinoma	1,419 (1013)		868 (529)		1,586 (829)		1900 (809)	
Others	1,154 (939)		862 (411)		1,165 (988)		1900 (887)	
Metastasis at diagnosis		**0.041**		0.69		**0.018**		**0.029**
Yes	1,349 (862)		872 (582)		1,381 (966)		1753 (807)	
No	1,449 (1002)		883 (489)		1,613 (756)		1959 (754)	
No. of clinical visits per patient		0.507		**0.035**		**<0.001**		**0.015**
1	1,436 (1099)		935 (570)		1900 (841)		2034 (689)	
2	1,424 (1113)		759 (413)		1,566 (757)		1925 (1087)	
3	1,377 (545)		793 (592)		1,411 (591)		1948 (997)	
4+	1,398 (770)		862 (387)		1,250 (523)		1789 (750)	
Treatment regimens		**<0.001**		**<0.001**		**<0.001**		**<0.001**
Chemotherapy alone	1,165 (778)		701 (555)		1,093 (688)		1,544 (643)	
Surgery alone	1770 (928)		1,125 (592)		2006 (698)		2,126 (678)	
Surgery combined with chemotherapy	1,289 (914)		839 (439)		1,432 (422)		1961 (728)	

CNY, China Yuan; IQR, interquartile range; Guangxi, Guangxi Zhuang Autonomous Region. Bold values indicate statistical significance (*p* < 0.05).

#### 3.4.2 Temporal trend of the medical expenditure per day

**Figure 6:**
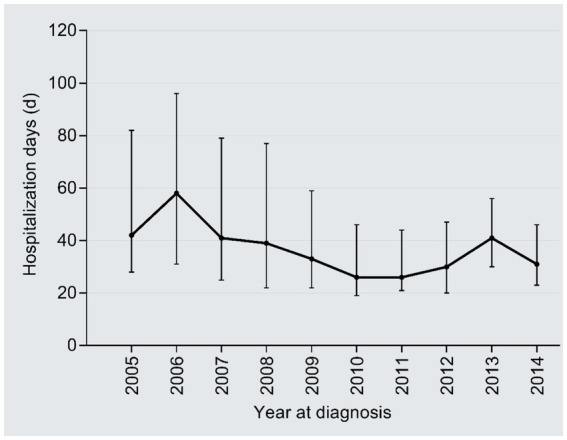
Temporal trend of the inpatient days.

**Figure 7:**
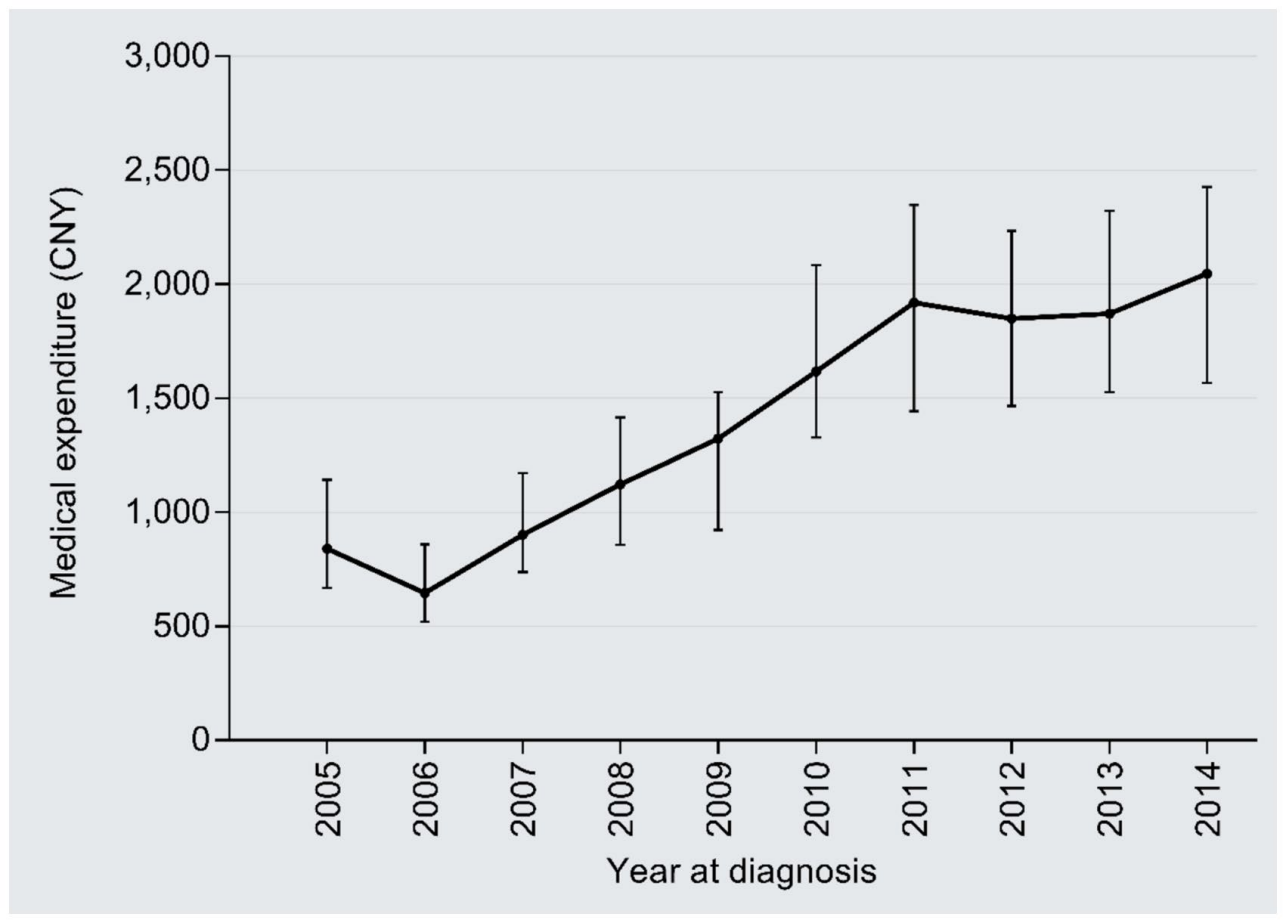
Temporal trend of the medical expenditure per day.

The inpatient days reduced from 42 days in 2005 to 31 days in 2014 (*r* = −0.18, 95%CI: −0.24, −0.12; *P* trend<0.001), with an AAPC of −3.32% (Figure 6). By contrast, the medical expenditures per day dramatically increased from 839 CNY in 2005 to 2,047 CNY in 2014 (2.44 times; *r* = 0.69, 95%CI:0.02–0.66, *P* trend<0.001), with an AAPC of 10.42% (Figure 7); the AAPC values were 10.19% in 2005–2008, 20.50% in 2009–2011, 5.18% in 2012–2014.

### 3.5 Sensitivity analysis

The results of the subgroup consistency tests are summarized in Table 7. The upward temporal trend in medical expenditure per patient observed in the overall cohort (AAPC: 5.59%) was consistently positive across all investigated patient subgroups. The AAPC estimates ranged from 4.82 to 6.15%, demonstrating remarkable homogeneity. Notably, the most substantial increases were observed among patients with health insurance (AAPC: 5.89%) and those diagnosed with stage I-II disease (AAPC: 6.15%). Similarly, the medical expenditure per day exhibited a pronounced and consistent increasing trend across all subgroups, with AAPCs ranging from 9.50 to 11.20%. The rapid growth in daily costs was universal, regardless of patient demographics, disease severity, or treatment received. In contrast, the trend for medical expenditure per clinical visit/admission, which showed a modest overall increase (AAPC: 1.41%), displayed greater variability across subgroups. While the direction of change was generally positive or neutral, the magnitude of the AAPC varied, from a slight decrease of −0.45% in the surgery-alone treatment subgroup to an increase of 3.02% in female patients.

**Table 7:** Average annual percent change (AAPC) of medical expense across patient subgroups (2005–2014).

Subgroup	Category	AAPC, Per Patient (%)	AAPC, Per Admission (%)	AAPC, Per Day (%)
Overall		5.59	1.41	10.42
Gender	Male	5.52	0.95	10.35
	Female	5.7	3.02	10.61
Age Group	<45 years	5.21	1.88	9.5
	45–54 years	5.88	1.12	10.85
	55–64 years	5.45	0.67	10.4
	≥65 years	5.81	1.75	11.2
Health insurance status	Had insurance	5.89	1.92	10.78
	No insurance	4.82	0.51	9.95
Cancer Stage	I-II	6.15	2.15	10.9
	III-IV	5.38	0.89	10.25
Treatment Regimen	Chemotherapy Alone	5.1	2.45	10.1
	Surgery Alone	5.65	−0.45	11.05
	Surgery + Chemotherapy	5.83	1.82	10.50

The sensitivity analysis confirmed that the core findings of rapidly increasing per-patient and per-day costs were robust and not driven by the specific distribution of characteristics within our sample. The consistent trends across diverse patient strata strengthen the validity of our primary conclusions regarding the escalating economic burden of CRC care in Guangxi during the study period.

## 4 Discussion

This study provides a comprehensive analysis of medical expenditures associated with the diagnosis and treatment of CRC in Guangxi between 2005 and 2014. The results indicate sustained and rapid growth in medical costs—whether measured per patient, per admission, or per day—alongside a corresponding decrease in the average length of hospital stay. Notably, the average annual medical expense per patient was 2.2 to 3.3 times the local per-capita GDP during the study period, highlighting a substantial and increasing economic burden. Multiple factors were significantly associated with medical expenditures, including treatment regimens, the number of clinical visits or admissions, and the number of hospitalization days.

### 4.1 Analysis of medical expenditure trends and economic burden

#### 4.1.1 Trends in medical expenditure

Between 2005 and 2014, medical expenses for CRC patients in Guangxi showed a pronounced upward trajectory, reflecting growing clinical demands and evolving treatment paradigms. This trend aligns with national data indicating a significant rise in cancer-related medical expenses in China, where total cancer care costs increased markedly from 63.30 billion CNY in 2008 to 249.56 billion CNY in 2017, with an annual percentage change (APC) of 15.2% (28). The growth pattern of CRC medical costs in Guangxi is consistent with national observations, where the average medical cost per patient more than doubled (23, 24).

#### 4.1.2 Comparison of medical expenditure with local economic level

In Guangxi, the total medical expenditure per patient reached 50,540 CNY, which was 2.6 times the local per capita GDP (19,469 CNY). Furthermore, the annual growth rate of per-patient medical costs (5.59%) exceeded the growth rate of per capita GDP (5.35%), underscoring healthcare inflation as an independent economic pressure point. The phenomenon of medical cost growth outpacing GDP growth has been confirmed by studies in multiple countries and regions. For example, in Europe, pharmaceutical spending for CRC increased by 213.7% between 2009 and 2015, far exceeding GDP growth, with healthcare inflation being a major driver of overall medical expenditure (29). Research in China also indicates that the economic burden of CRC reached 170.5 billion CNY in 2019, accounting for 0.189% of the local GDP, and this burden is projected to continue rising without effective interventions (30).

Although economic development promotes the quality of medical services and technological advancement, it simultaneously exacerbates the economic burden on patients. As economic levels improve, more high-priced new drugs and advanced treatment regimens enter clinical practice. For instance, in China’s third-line therapy, while fruquintinib may offer cost savings compared to regorafenib, its high cost still imposes pressure on patients and the healthcare insurance system (31). Additionally, economic development increases the demand for medical services, with patients seeking higher-quality healthcare resources, further driving up medical costs (32). Consequently, despite the upgrades in medical services facilitated by economic growth, the economic burden on patients has not been effectively alleviated and has instead intensified due to costs rising faster than GDP.

#### 4.1.3 Regional variations in medical expenditure

During 2009–2011, medical expenses for CRC patients in Guangxi (47,842 CNY) were slightly lower than the national average (48,163 CNY) and the Eastern region’s average (52,390 CNY), comparable to the Central region (47,294 CNY), but significantly higher than the Western region’s average (38,108 CNY) (22). These disparities reflect differences in hospital type, grade, and regional economic development levels. Costs are typically higher in specialized hospitals and Grade 3A hospitals (22). Subgroup analyses revealed that costs for female patients, those with stage III disease, and those undergoing surgical treatment in Guangxi exceeded the national average, whereas costs for male patients, those with stage IV cancer, and those receiving chemotherapy alone were lower.

Hospital grade and type are important factors influencing medical costs. Higher-level hospitals possess more advanced equipment and specialized staff, undertake more complex treatments, and generally have higher costs (33). Regional economic development levels directly affect hospital service capacity and pricing standards (34). Furthermore, patient gender, disease stage, and treatment regimen choices also contribute to cost variations, with advanced disease and comprehensive treatments typically associated with higher expenditures (35, 36).

### 4.2 Clinical characteristics of colorectal cancer patients and regional differences

#### 4.2.1 Regional features in sex ratio and age distribution

The male-to-female ratio among CRC patients in Guangxi (1.54:1) was higher than the national average (1.34:1) (37), and the mean age at diagnosis (57.25 years) was notably younger (38). The higher incidence in males may be associated with lifestyle risk factors such as smoking, alcohol consumption, and obesity (39, 40). The association between metabolic syndrome, non-alcoholic fatty liver disease, and CRC risk is more pronounced in males (41). The younger age at diagnosis aligns with the global trend of rising early-onset CRC incidence (42, 43). Tumors in younger patients are often located in the distal colon and rectum, exhibit poorer histological differentiation, and demonstrate more aggressive biological behavior (44).

#### 4.2.2 Diagnostic staging and screening status

The proportion of late-stage diagnoses (Stage III-IV) was as high as 60%, far exceeding that of early-stage cases (Stage I, only 4%). This disparity primarily stems from the lack of organized population-based screening programs (8, 24) and the influence of health insurance policies (45, 46). The lower costs for patients diagnosed at an early stage (24) highlight the economic value of improving the screening system. Imbalanced distribution of medical resources, limited diagnostic capacity at the primary care level, and inconsistencies between clinical and pathological staging further impede early diagnosis rates.

#### 4.2.3 Impact of health insurance policies and healthcare-seeking behavior

The expansion of health insurance coverage following the 2009 healthcare reform improved access to diagnostic and treatment services (47–50). Measures such as the 2017 national drug price negotiation helped control drug costs (51–54), yet affordability challenges persist. Reimbursement constraints often lead to delayed referrals to specialized centers for rural and low-income patients, resulting in late-stage diagnoses. Optimization of health insurance policies should focus on increasing reimbursement rates, streamlining reimbursement procedures, and establishing special subsidies for major diseases, while also guarding against overutilization (55–59).

### 4.3 Multivariate analysis of factors influencing medical costs

#### 4.3.1 Impact of diagnostic technology and treatment regimens on costs

The increase in medical expenditure can be attributed to several interrelated factors, including the wider application of advanced diagnostic technologies (24), the introduction of novel—though often costly—anticancer drugs (60–62), and the use of comprehensive treatment regimens such as surgery combined with chemotherapy, which was associated with the highest costs in this study. Existing research indicates that advanced diagnostic technologies such as CT colonography, molecular diagnostics, and AI-assisted diagnosis improve diagnostic accuracy but also increase medical costs (63–65). Treatment regimens involving surgery combined with chemotherapy are the most expensive, and comprehensive treatment models significantly increase total expenditure (32, 66). Although targeted therapies improve outcomes, their high cost adds to the economic burden (67, 68). Clinical decision-making must balance treatment efficacy with economic sustainability, promoting precision medicine and rational drug use.

#### 4.3.2 Dominance of drug costs

Although targeted therapies have improved clinical outcomes, their high prices have significantly increased the financial burden on patients (69). A pivotal finding relates to the structure of medical expenditure, which is dominated by drug fees (61.22%). This aligns with patterns reported in prior studies across China, where drug costs typically constitute 40–60% of direct cancer care expenses (32). This “drug-centered” cost structure, accompanied by disproportionately low technical service fees (e.g., for nursing and treatment), reflects historical distortions in the medical service pricing system in China (70). Since the conclusion of our study period, China has implemented profound healthcare reforms—including the zero-markup drug policy and adjustments to medical service prices—which may help rebalance the expenditure structure in the future. Further research is essential to evaluate the impact of these reforms on reducing the share of drug expenditure and appropriately valuing healthcare professionals’ labor.

### 4.4 Limitations

This study has several limitations. First, as a single-center study conducted in a tertiary hospital, the findings are representative of specialized, high-level cancer care settings in Guangxi but may not be broadly generalizable to patients in primary care or other regions. The sample had a higher male proportion and younger age at diagnosis compared to national averages, which might introduce selection bias. However, sensitivity analyses (e.g., subgroup consistency and weight adjustment) showed that the key trends were robust across different patient characteristics. Second, cost data were obtained solely from our institution, and some patients had prior diagnoses or treatments elsewhere, potentially leading to underestimation of total expenses. Third, the reliability of results depends on the completeness and accuracy of medical records.

Despite these limitations, as part of the health economics evaluation of the Cancer Screening Program in Urban China, this study offers several important practical implications. It provides a comprehensive overview of actual medical expenditures for CRC diagnosis and treatment, including decadal trends and influencing factors; supplies valuable evidence to inform the design of future CRC screening programs in urban Guangxi; and establishes a useful baseline for subsequent research in Guangxi, the broader southwestern region, and other comparable provinces in China.

## 5 Conclusion

This study demonstrates that the economic burden of CRC in Guangxi from 2005 to 2014 was substantial and grew rapidly, outpacing local economic growth. The median medical expenditure per patient was 2.6 times the local per-capita GDP, with costs driven significantly by a high proportion of late-stage diagnoses (59.87% at Stage III–IV), intensive treatment regimens such as surgery combined with chemotherapy, and prolonged hospital stays. Critically, the cost structure was dominated by pharmaceutical expenses, which accounted for 61.22% of total expenditures—reflecting systemic distortions in the historical pricing of healthcare services.

These findings underscore the urgent need for integrated, multi-level policy interventions to curb cost escalation and improve the efficiency and equity of CRC care. Specifically, we recommend:

First, establishing organized, population-based screening programs is fundamental to addressing the high proportion of late-stage diagnoses (59.87% at Stage III-IV) identified in this study. Prioritizing screening resources for high-risk groups can significantly improve early detection rates, thereby avoiding the high-intensity, high-cost treatments associated with advanced disease (71, 72). This is particularly crucial for regions like Guangxi, where per-patient medical expenses were a heavy multiple of the local per-capita GDP. Substantial evidence confirms that such screening is cost-effective and can reduce the direct and indirect economic losses from late-stage treatment (73, 74).

Second, optimizing the allocation of medical resources is key to controlling the unchecked growth of per-patient costs. Our study identified treatment modalities (e.g., surgery combined with chemotherapy) and the length of hospital stay as significant cost drivers. Establishing a tiered healthcare delivery system, which enhances the capacity of primary care institutions for screening, risk assessment, and patient navigation (75, 76), and fosters collaboration between tertiary and specialized hospitals, can optimize patient pathways and prevent the over-concentration and waste of resources. Promoting optimized protocols such as minimally invasive surgery and Enhanced Recovery After Surgery (ERAS) can help shorten the extended hospital stays observed in our study, thereby freeing up medical resources (68, 77).

Third, deepening health insurance reforms and price controls are imperative to directly address the most prominent issue in our cost structure: the dominance of drug expenditures (61.22%). On one hand, insurance coverage should be expanded for preventive services, such as waiving out-of-pocket costs for CRC screening, which has proven effective in increasing participation rates (78). On the other hand, robust national drug price negotiations and the promotion of biosimilars are central strategies for controlling anticancer drug costs and rectifying the distorted expenditure structure historically driven by the “drug markup” policy (79, 80). Payment model reforms should also support efficient models like multidisciplinary care and provide targeted protection for vulnerable groups such as the older adults and low-income populations (81, 82).

This study establishes a critical baseline for assessing the impact of subsequent health reforms in Guangxi and comparable regions. Future research should adopt multicenter designs and incorporate recent data to evaluate reform effectiveness and further refine CRC control strategies.

## Data Availability

The original contributions presented in the study are included in the article/supplementary material, further inquiries can be directed to the corresponding author.
